# Does medial branch radiofrequency neurotomy accelerate degenerative lumbar spondylolisthesis compared to natural progression? A cross-sectional cohort study

**DOI:** 10.1016/j.inpm.2023.100289

**Published:** 2023-11-01

**Authors:** Marc Caragea, Austin Le, Tim Curtis, Amelia Ni, Tyler Clark, Andrew Joyce, Colton Hickman, Brandon Lawrence, Zane Randell, Perry Goodman, Addisyn Poduska, Michaela Rasmussen, Amanda Cooper, Masaru Teramoto, Taylor Burnham, Aaron Conger, Zachary L. McCormick

**Affiliations:** a Department of Physical Medicine and Rehabilitation, University of Utah Health, Salt Lake City, UT, USA; b Department of Orthopedics, Division of Physical Medicine and Rehabilitation, Washington University in St. Louis, St. Louis, MO, USA; c School of Medicine, University of Utah, Salt Lake City, UT, USA

**Keywords:** Facet, Joint, Back pain, Outcomes

## Abstract

**Background:**

Lumbar radiofrequency neurotomy (LRFN) effectively treats lumbar zygapophyseal joint pain by coagulating medial branch nerves (MBNs) carrying nociceptive signals. MBNs also innervate deep paraspinal muscles. There is a paucity of literature on whether LRFN accelerates the progression of vertebral displacement in patients with degenerative lumbar spondylolisthesis (DLS).

**Objective:**

Compare the rate of spondylolisthesis progression in adults with DLS who underwent LRFN to the 2% annual rate of progression expected by natural history.

**Design:**

Cross-sectional cohort study.

**Methods:**

Consecutive patients with pre-existing DLS who underwent LRFN for zygapophyseal joint-mediated low back pain were identified. Patient demographics, LRFN procedure details, and radiographic images confirming Meyerding Grade (I-II) spondylolisthesis were collected from electronic medical records. The quantitative magnitude of spondylolisthesis progression and the annualized rate were calculated from pre-and post-LRFN radiographs. Data were analyzed using Wilcoxon signed-rank tests and a linear regression model.

**Results:**

152 patients (mean age 65.9 ± 12.3 years; 59.2% female) met eligibility criteria and were included in the analyses. Average time to radiographic follow-up was 35.6 ± 24.7 months post-LRFN. The average spondylolisthesis progression rate of 1.63 ± 2.91% per year calculated for the LRFN cohort was significantly lower than the 2% annual rate of progression associated with natural history (*p* < 0.001). None of the included covariates, such as age, BMI, LRFN laterality, number of levels denervated, or history of prior lumbar spinal surgery, were significantly associated with the average annual rate of progression.

**Conclusions:**

Our results suggest that spondylolisthesis progression rate is no different or worse than the expected natural progression rate in patients with pre-existing DLS who have undergone LRFN.

## 1 Introduction

Spondylolisthesis is characterized by the displacement or slippage of one vertebral body over another. Degenerative lumbar spondylolisthesis (DLS) is a specific type of spondylolisthesis that occurs due to age-related spondylotic changes in the lumbar spine and features an intact neural arch [1,2]. Degenerative lumbar spondylolisthesis is commonly observed at the L4-5 level and tends to affect women more frequently than men. While it is uncommon in adults younger than 50 years of age, the prevalence increases as individuals progress into their eighth decade [[3], [4], [5], [6], [7]].

DLS is also associated with progression by natural history on a population-level. One-third of adults with pre-existing DLS will experience disease progression, defined as 5% or greater displacement [8]. In this subset of patients, the rate of progression is estimated to be 2% per year [9,10]. Lack of progression in some individuals and slow progression in others is partially due to the stabilizing effects of vertebral osteophyte formation, cartilaginous endplate sclerosis, degenerative zygapophyseal joint hypertrophy, and ligamentous ossification in advanced disease [11]. As demographics in the United States shift towards an aging population, it is important to understand factors which may affect the progression of degenerative spondylolisthesis. For patients with a history of neurogenic claudication or radiculopathy, degenerative changes associated with spondylolisthesis progression often necessitate surgical decompression and fusion to alleviate neurologic symptoms and stabilize the affected spinal unit [12]. According to national trends from 2004 to 2015, rates of elective lumbar fusion operations and associated hospital costs are increasing the fastest among adults aged 65 years and older, with degenerative spondylolisthesis accounting for the majority (45%) of fusion surgeries in 2015 [13]. In addition to various operative and peri-operative risks, lumbar fusion is accompanied by a long-term possibility of re-operation to address known complications from adjacent segment degeneration [14]. Thus, total health care expenditures are not limited to significant hospital costs for an inpatient surgery, but also include services required during the complete episode of care.

While there is a lack of direct correlation between DLS and low back pain, the contribution of zygapophyseal joint pathology to DLS and its impact on function and quality of life make it an important area of focus for treatment [5,6,8,10]. Intuitively, displacement of a spinal motion segment disrupts biomechanical loading patterns within both the anterior and posterior spinal elements. Zygapophyseal joint pain is thought to be the dominant pain generator in 15–40% of cases of LBP [5,15], though the prevalence rate in individuals with DLS and LBP is not well defined.

Lumbar radiofrequency neurotomy (LRFN) is a well-established, safe, and effective method for treating lumbar zygapophyseal joint pain by coagulation of lumbar medial branch nerves to disrupt nociception [[16], [17], [18], [19], [20], [21], [22]]. Because the lumbar multifidi are innervated, in part, by the lumbar medial branch nerves, denervation and subsequent atrophy of the lumbar multifidus muscle has been proposed as a theoretical risk of LRFN. However, no studies to date have definitively confirmed this adverse outcome [23,24]. As the multifidi muscles play an essential role in postural spinal stability [25], there is a theoretical concern that LRFN can lead to increased segmental instability and accelerate degenerative changes, particularly in patients with pre-existing DLS. Despite limited research on this topic, recent evidence suggests that LRFN does not significantly increase the rate of progression in DLS compared to the expected rate of progression in the general population [26].

This study reports the rate of spondylolisthesis progression in adults with DLS who underwent LRFN and compares it to the progression rate of 2% per year associated with natural history [9,10].

## 2 Methods

### 2.1 Data collection

This single-center cross-sectional cohort study was approved by the University of Utah Institutional Review Board (IRB 00138414). Electronic medical records of consecutive patients who underwent LRFN from July 2014–May 2020 were reviewed. Patient inclusion criteria were 1) age ≥18 years with pre-existing DLS; 2) LRFN for the treatment of zygapophyseal joint-mediated low back pain as confirmed by dual diagnostic blocks; 3) baseline neutral, standing lateral X-rays displaying Meyerding Grade (I-II) spondylolisthesis within one year before LRFN; and 4) corresponding X-rays within 6 months–10 years post-LRFN to evaluate the degree of DLS progression. Patients were excluded for having Meyerding Grade III-IV spondylolisthesis, lacking pre-and/or post-procedure weight-bearing lateral x-rays of the lumbar spine, having pars interarticularis defect(s) noted on imaging, or undergoing post-LRFN spinal fusion surgery. While patients were not excluded for having pre-LRFN spinal surgeries, including fusions, levels with a fusion were excluded and radiographic analysis was restricted to adjacent and non-fused segments only.

### 2.2 Radiographic evaluation

Pre- and post-procedural radiographs were used to measure the magnitude of spondylolisthesis and annualized rate of progression at levels treated with LRFN according to previously-described methods [27]. For individuals with spondylolisthesis at multiple levels, the level with the highest percentage of progression was selected for analysis. Spondylolisthesis was quantified in millimeters by determining the displacement of the caudal endplate of the superior vertebral body relative to the adjacent inferior vertebral body. The annual progression rate was then calculated using the following formula:

[(Post RFN displacement mm − Pre RFN displacement mm)/Endplate Distance mm] × 100% × (12 Months/Months Follow-up)

Radiographic measurements were performed by three study authors (MC, TC, and CH). Each radiograph was independently reviewed by two individuals, with any disagreements resolved by a third reviewer.

### 2.3 Procedures

All LRFN procedures were performed by physician specialists in Physical Medicine and Rehabilitation with fellowship training in either Pain Medicine, Sports Medicine, or Interventional Spine and Musculoskeletal Medicine.

### 2.4 Statistical analysis

The primary outcome measure was the average annual progression rate of spondylolisthesis expressed as a percentage (calculated as the highest value across all levels treated by LRFN per patient). Descriptive statistics were calculated for patient demographic variables. A one-sample Wilcoxon signed-rank test was used to compare the average annual progression rate for patients who received LRFN with the reference value of 2.0% [9,10]. Lastly, a linear regression model was created using robust standard errors to explore the relationship between the average annual progression rate with selected covariates.

## 3 Results

A total of 152 patients met criteria for inclusion and were included in the analyses, with a mean age of 65.9 years and a mean BMI of 29.9. Over half (59.2%) of the patients were female. The average time of X-ray follow-up post-LRFN was 35.6 ± 24.7 months. Of the 152 patients, 51 (33.6%) underwent a unilateral LRFN, while 101 (66.4%) underwent a bilateral LRFN procedure. Most patients (68.4%) had two levels denervated, while 27.0% had one level, and only 4.6% had three levels denervated. A total of 23 patients (15.1%) had a history of lumbar spinal fusion, and 17.8% had a history of non-fusion lumbar surgery. Patient demographics are presented in Table 1a and b.

**Table 1:** Patient demographics and clinical characteristics (*N* = 152).

(a)		
Continuous Variable	Mean (SD)	Min, Max

Age (years)	65.9 (12.3)	29.0, 96.0
BMI	29.9 (6.9)	14.6, 53.3
Follow-up (months)	35.6 (24.7)	6.0, 113.0

(b)		
Categorical Variable	Frequency	%
Gender		
Male	62	40.8
Female	90	59.2
LRFN laterality		
Unilateral	51	33.6
Bilateral	101	66.4
Number of levels denervated		
One	41	27.0
Two	104	68.4
Three	7	4.6
History of non-fusion lumbar spine surgery prior to LRFN		
Yes	27	17.8
No	125	82.2
History of lumbar spine fusion prior to LRFN		
Yes	23	15.1
No	129	84.9

Max = maximum value; Min = minimum value; SD = standard deviation.

LRFN = lumbar radiofrequency neurotomy.

**Fig. 1:**
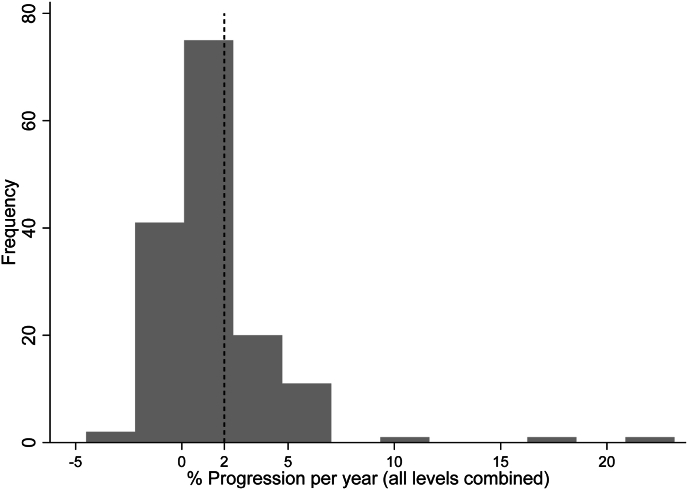
Histogram of % progression per year (all levels combined, using values from the level with the greatest displacement for patients with more than one affected level). Note: Dashed line represents the 2.0% reference value for annual % progression.

The results showed that the average percent progression per year was 1.63 ± 2.91%, which was significantly lower than the reference value of 2.0% (*p* < 0.001; Table 2 and Fig. 1). No significant associations were found between the average percent progression per year and any of the covariates such as age, BMI, laterality of LRFN, number of levels denervated, or history of lumbar spinal surgery (*p* > 0.05; Table 3).

**Table 2:** Spondylolisthesis percent progression per year by vertebral level (*N* = 152).

% Progression per Year	*N*	Mean (SD)	Median (IQR)	Min, Max	95% CI	*p*^a^
Vertebral Level						
L1-2	32	0.12 (2.66)	0.00 (0.00, 0.46)	−7.89, 10.08	−0.84, 1.08	**< 0.001**
L2-3	57	0.71 (3.53)	0.00 (−0.47, 1.08)	−4.94, 23.18	−0.23, 1.65	**< 0.001**
L3-4	58	0.10 (2.04)	0.00 (−0.96, 1.2)	−6.72, 4.18	−0.44, 0.64	**< 0.001**
L4-5	81	0.91 (2.94)	0.64 (0.00, 1.86)	−9.97, 18.02	0.26, 1.56	**< 0.001**
L5-S1	60	0.90 (2.36)	0.84 (0.00, 2.02)	−6.32, 6.06	0.29, 1.51	**< 0.001**
All levels combined^b^	152	1.63 (2.91)	1.01 (0.00, 2.03)	−4.51, 23.18	1.16, 2.10	**< 0.001**

CI = confidence interval; IQR = interquartile range; Max = maximum value; Min = minimum value; SD = standard deviation.

a From significance test (vs. 2.0%), using one-sample Wilcoxon signed-rank test.

b Using highest spondylolisthesis progression value across all levels per patient.

**Table 3:** Linear regression model on percent progression per year (all levels combined) by covariates.

Covariate	B	95% CI	*p*
BMI	0.03	−0.06, 0.13	0.465
Age	0.02	−0.02, 0.07	0.256
LRFN laterality (vs. unilateral)			
Bilateral	0.79	−0.14, 1.71	0.096
Number of levels denervated (vs. one)			
Two	0.48	−1.05, 2.00	0.537
Three	−0.29	−1.81, 1.23	0.704
History of non-fusion lumbar spine surgery prior to LRFN (vs. no)	−0.23	−1.45, 1.00	0.717
Yes			
History of lumbar spine fusion prior to LRFN (vs. no)	0.72	−2.30, 3.75	0.638
Yes	−1.91	−6.37, 2.54	0.397

B = beta coefficient; CI = confidence interval; LRFN = lumbar radiofrequency neurotomy.

Note: Outcome = % progression per year (all levels combined, with value from level that progressed the most).

## 4 Discussion

We report the rate of spondylolisthesis progression in a real-world clinical population of adults with DLS who underwent LRFN and compare it to the annual 2% progression rate associated with natural history [9,10]. In this cohort, we observed that vertebral displacement progressed at an average annual rate of 1.63 ± 2.91%, which was significantly lower than the 2% reference value. None of the covariates measured in this study, including aspects of patient demographics, clinical history, or LRFN procedure details, were significantly associated with average annual progression rate. These results suggest LRFN may have no clinically meaningful impact on the natural progression of vertebral displacement predicted to occur in patients with DLS over time.

The natural progression of DLS has not been extensively studied in the literature, and there is a scarcity of available data for comparison. In 1990, Matsunaga et al. conducted a study of 40 patients with DLS who had a minimum displacement of 5% using the Morgan and King compass method [8]. They found that the patients had an overall displacement range of 7–28% at the initial examination, with most participants falling in the 10–19% range. The progression of displacement was defined as an increase of 5% or more, as assessed by lateral lumbar radiographs. The follow-up duration was 5–14 years, with an average of approximately 8 years. Using this definition, spondylolisthesis progression was observed in 30% of subjects. In a subsequent study, Matsunaga et al. observed 157 non-surgically managed DLS patients for an average period of roughly 16 years (ranging between 10 and 18 years) and noted spondylolisthesis progression in 34% of participants using the same 5% threshold [9]. Similarly, Denard et al. performed a prospective study of men over 65 and found a similar progression range (5–28%) on initial radiologic evaluation in a subpopulation of subjects with pre-existing DLS [10]. However, they noted a lower prevalence of spondylolisthesis progression: only 12% of those with displacement at baseline exhibited progression (ranging from 5 to 10%) at an average follow-up of 4.6 years. Denard et al. combined their data with Matsunaga et al. to show that spondylolisthesis progresses at approximately 2% per year [9,10]. However, these studies did not comment on whether the spondylolisthesis progression occurred at a constant pace or if a maximum displacement threshold was rapidly achieved and stabilized.

Later, Cushnie et al. conducted a prospective study in 160 adult patients with non-surgically managed DLS to examine functional outcome measures and displacement progression [28]. The study was similar to Matsunaga et al. and used the 5% threshold for spondylolisthesis progression [9]. Over 5 years, 31.8% of the cohort showed progression. Although self-reported outcome measures such as 12-Item Short Form Physical Component Score (SF12-PCS), Oswestry Disability Index (ODI), and leg pain improved for both the progressive and non-progressive DLS subtypes, back pain only increased in the non-progressive subtype.

The present study differed from the previously mentioned research on the progression of degenerative lumbar spondylolisthesis in several ways. Firstly, it did not exclude patients who showed less than 5% spondylolisthesis progression, providing a broader sample compared to earlier studies. Furthermore, it evaluated the impact of LRFN on the progression of spondylolisthesis over a longer period. Patel et al. conducted a small observational study with 14 patients with existing DLS who underwent LRFN and evaluated the advancement of their spondylolisthesis over time [26]. These investigators found that the percent progression of spondylolisthesis per year was 1.30% (95% CI −0.14 to 2.78%) with a mean follow-up time of 23.9 months. However, these results were limited by the small sample size and relatively short follow-up time, possibly failing to detect delayed progression.

The effect of LRFN on the progression of spondylolisthesis is a topic of interest due to the potential for denervation and subsequent atrophy of stabilizing lumbar multifidi muscles. The multifidi muscles form the posterior portion of the inner muscular ring of the spine, and their atrophy could lead to spinal destabilization [29]. Similarly, the longissimus and iliocostalis muscles may also be at risk of damage if adjacent branching nerves from the common dorsal ramus are inadvertently affected during LRFN [30]. Despite the theoretical risk of spinal destabilization as an adverse effect of LRFN, we found no evidence supporting this outcome in our cohort of adults with pre-existing DLS.

Our results also showed a statistically significantly lower spondylolisthesis progression rate than the typical 2% natural progression rate. While it is impossible to conclude that LRFN has a direct protective effect, these results still have important clinical implications. LRFN has proven to be an effective treatment for patients with zygapophyseal joint-mediated pain and can continue to provide benefits for many patients with DLS without significantly impacting the progression of spondylolisthesis.

## 5 Limitations

The results of our study should be interpreted with caution as they come from a single institution and may not be representative of other clinical practices or patient demographics. The findings reflect the specific circumstances and characteristics of the institution and may not apply or be generalizable to different settings. Furthermore, the study's cross-sectional design also poses inherent limitations that should be considered when interpreting the results. Because lumbar flexion/extension x-rays were not available for many patients both before and after the index LRFA procedure, we used static standing films only. As such, our findings do not account for the possibility of new dynamic instability. While we evaluated radiographs using an established method for determining the magnitude and annual progression rate of spondylolisthesis, other techniques may be more accurate [27].

The reference rate of 2% progression per year used in our study was obtained from the analysis of previous studies on progression in adults with DLS. However, further research is necessary to fully determine this reference rate's validity. Notably, the magnitude of vertebral displacement reported by Matsunaga and Denard et al. at initial examination was consistent with Meyerding grade I spondylolisthesis in 100% (mean displacement 13.6%; range 7–20%) and 99% (displacement range 5–28%) of patients with pre-existing DLS, respectively, suggesting the 2% annual progression rate was derived exclusively from cases of low-grade spondylolisthesis [9,10]. The generalizability of the present study's findings is similarly limited by our decision to include only patients with Meyerding grade I-II spondylolisthesis. Restricting enrollment to this patient subpopulation makes sense in light of the research question of whether RFN causes DLS to progress more rapidly than would be expected by natural history alone. Given the historical uncertainty regarding the relationship between LRFN and multifidus muscle atrophy, we expect practitioners are more likely to pursue surgical treatments for low back pain in patients with advanced (Meyerding grade III-IV) DLS due to concerns of exacerbating instability with LRFN. However, further research is warranted to determine whether the natural rate of annual spondylolisthesis progression varies with the severity of vertebral displacement. A larger prospective cohort study with a longer follow-up period is required to understand the effects of LRFN more accurately on spondylolisthesis progression. Additionally, there may be a nonlinear progression that should be taken into account. These findings emphasize the importance of additional research to gain a deeper understanding of the progression of DLS and its relationship with different patient factors.

Finally, the study cohort was comprised of patients who had undergone spinal surgery, including both fusion and non-fusion surgeries. Approximately 32.9% of patients underwent spinal surgical intervention before LRFN, with 15.1% having a fusion. Although spinal surgical intervention, particularly fusions, may have influenced the degree of adjacent segment spondylolisthesis progression in this subgroup, it was deemed essential to include these patients in the study to provide an accurate representation of the population that commonly seeks treatment for zygapophyseal joint-mediated pain.

## 6 Conclusion

Given the high prevalence of concomitant DLS and zygapophyseal joint pain, it is vital to consider the potential consequences of treating zygapophyseal joint pain with techniques such as LRFN on the progression of DLS. In this cohort, spondylolisthesis progressed more slowly after LRFN compared to its natural history. Although it is unlikely that LRFN has a beneficial protective effect on the advancement of spondylolisthesis, the results of the present study suggest that the annual progression rate of spondylolisthesis at levels treated with LRFN is no different or worse than that expected by natural history alone.

## Funding

This study was funded by (1) the Skaggs Clinical Spine Research Seed Grant Program at the 10.13039/100007747University of Utah, and (2) the 10.13039/100007747University of Utah June Morris Spine Research Grant.

## Declaration of competing interest

Zachary L. McCormick, MD has received research funding from Avanos Medical (paid directly to the 10.13039/100007747University of Utah) and serves on the Board of Directors of the Spine Intervention Society (SIS). Aaron Conger, DO has received research funding from Stratus Medical (paid directly to the 10.13039/100007747University of Utah). Taylor Burnham, DO MS has received research funding from DIROS Technology (paid directly to the 10.13039/100007747University of Utah). There are no other potential conflicts of interest to disclose on the part of any of the other authors.
